# Supplementary material for: No evidence for manipulation of Anopheles gambiae, An. coluzzii and An. arabiensis host preference by Plasmodium falciparum
Source: Sci Rep. 2017 Aug 25;7:9415. doi: 10.1038/s41598-017-09821-x (PMC5572726; doi:10.1038/s41598-017-09821-x)
Supplement: Supplementary file 1 — Supplementary material [file 41598_2017_9821_MOESM1_ESM.pdf]

**No evidence for manipulation of *Anopheles gambiae*, *An. coluzzii* and *An. arabiensis* host preference by *Plasmodium falciparum***

Phuong L. Nguyen, Amélie Vantaux, Domonbabele FdS Hien, Kounbobr R. Dabiré, Bienvenue K. Yameogo, Louis-Clément Gouagna, Didier Fontenille, François Renaud, Frédéric Simard, Carlo Costantini, Fréderic Thomas, Anna Cohuet, Thierry Lefèvre

**Supplementary Figure S1.** Parasite prevalence (proportion of infected females ± 95% confidence interval) at the oocyst stage of the three mosquito species. Letters below the bar indicate the gametocyte carrier and its gametocytemia.

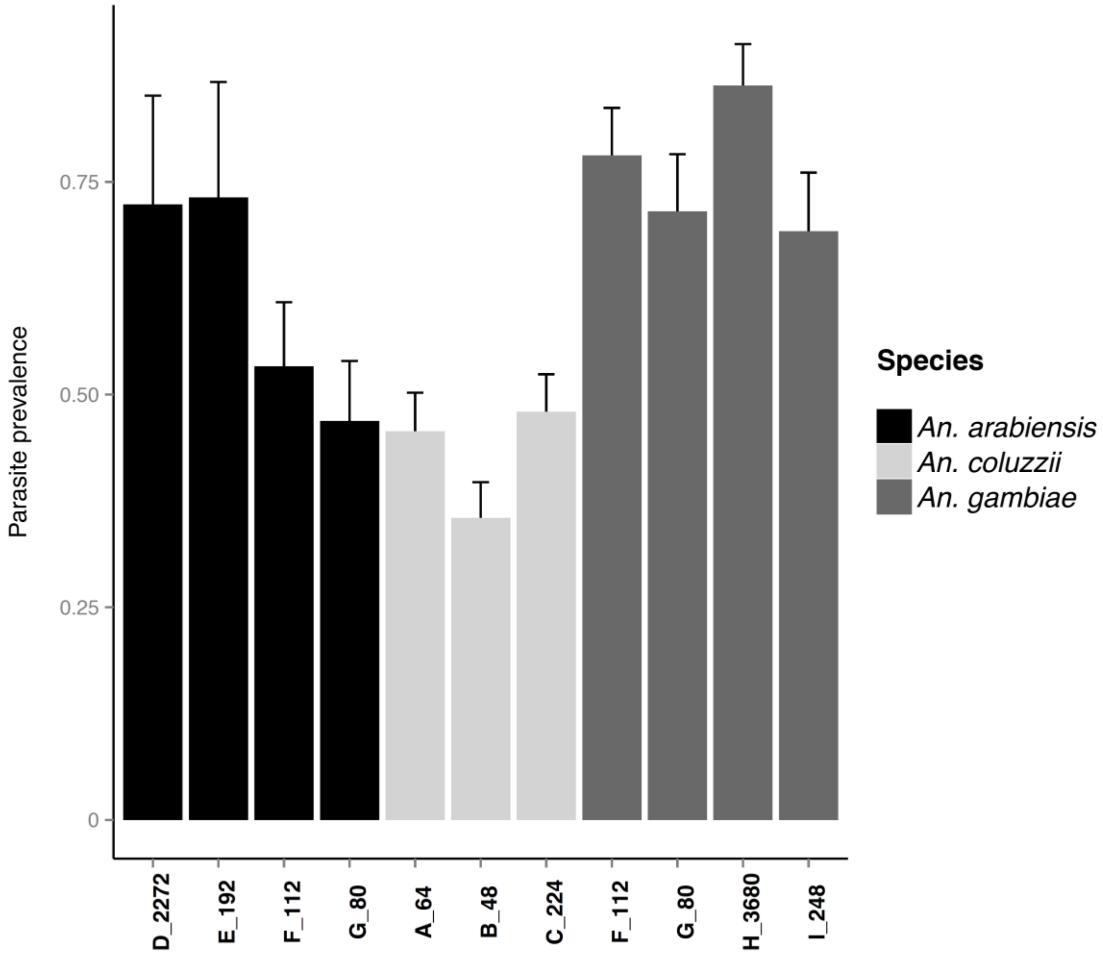

**Supplementary Figure S2.** Parasite intensity (mean number of parasites in the midgut of infected females ± se) at the oocyst stage of the three mosquito species. Letters below the bar indicate the gametocyte carrier and its gametocytemia.

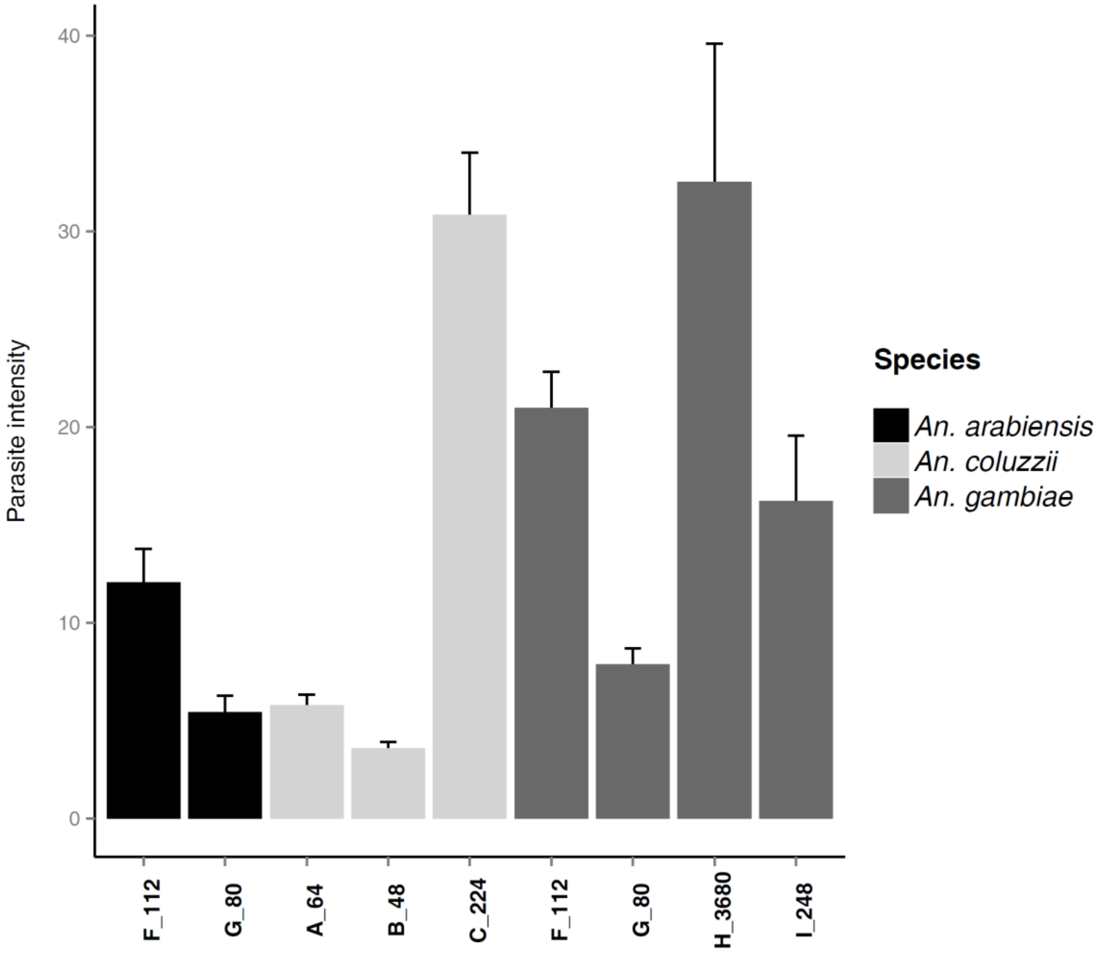

**Supplementary Table S1.** Details of carrier, gametocytemia, test period and total number of behavioral runs for each replicate

| Species | Experimental replicate | Carrier | Gametocytes/ µl | Mean oocyst (Range) | Test period | Total runs |
|---|---|---|---|---|---|---|
| *Anopheles coluzzii* | 1 | A | 64 | 5.79 (1 - 23) | Test period 1 (6, 7, 8 dpi) | Test period 1: |
| | | | | | Test period 2 (13, 14, 15 dpi) | H-C: 18 |
| | 2 | B | 48 | 3.59 (1 – 17) | Test period 1 (6, 7, 8 dpi) | H-O: 18 |
| | | | | | Test period 2 (13, 14, 15 dpi) | Test period2: |
| | 3 | C | 224 | 30.85 (1 - 163) | Test period 1 (6, 7, 8 dpi), | H-C: 18 |
| | | | | | Test period 2 (13, 16, 17 dpi) | H-O: 18 |
| *Anopheles arabiensis* | 1 | D | 2272 | NA | Test period 2 (17 dpi) | Test period 1: |
| | 2 | E | 192 | NA | Test period 2 (17 dpi) | H-C: 2 |
| | 3 | F* | 112 | 12.06 (1 - 65) | Test period 1 (5, 6, dpi) | H-O: 2 |
| | | | | | Test period 2 (17, 18 dpi) | C-O: 2 |
| | | G* | 80 | 5.42 (1 - 34) | Test period 1 (5, 6, dpi) | Test period2: |
| | | | | | Test period 2 (17, 18 dpi) | H-C: 6 |
| | | | | | | H-O: 6 |
| | | | | | | C-O: 6 |
| *Anopheles gambiae* | 1 | F* | 112 | 20.99 (1 – 79) | Test period 1 (8, 9, dpi) | Test period 1: |
| | | | | | Test period 2 (17, 18 dpi) | H-C: 8 |
| | | G* | 80 | 7.88 (1 – 32) | Test period 1 (8, 9, dpi) | H-O: 8 |
| | | | | | Test period 2 (17, 18 dpi) | C-O: 8 |
| | 2 | H | 3680 | 32.53 (1 – 222) | Test period 1 (7, 8, dpi) | Test period2: |
| | | | | | Test period 2 (18, 19 dpi) | H-C: 8 |
| | | I | 248 | 16.21 (1 – 82) | Test period 1 (7, 8, dpi) | H-O: 8 |
| | | | | | Test period 2 (18, 19 dpi) | C-O: 8 |

* *indicates that the serum of the carrier was replaced by European serum*

dpi: day post infection

**GLMM models for two groups of mosquitoes: infected (exposed-infected) and uninfected controls (unexposed).**

**Supplementary Table S2: Effect of infection treatment, combination and test period on *Anopheles coluzzii* activation rate**

| Variable | LRT | df | *P* |
|---|---|---|---|
| Infection treatment | 0.50 | 1 | 0.48 |
| Combination | 3.21 | 1 | 0.07 |
| Test period | 0.08 | 1 | 0.78 |
| Test period:combination | 1.06 | 1 | 0.30 |
| Infection treatment:combination | 0.22 | 1 | 0.64 |
| Infection treatment:test period | 1.13 | 1 | 0.29 |

**Supplementary Table S3: Effect of infection treatment, combination, test period on *Anopheles gambiae* activation rate**

| **Variable** | **LRT** | **df** | ***P*** |
|---|---|---|---|
| Infection treatment | 0.22 | 1 | 0.64 |
| Combination | 3.26 | 2 | 0.20 |
| Test period | 3.94 | 1 | **0.047 *** |
| Combination:test period | 8.44 | 2 | **0.01*** |
| Infection treatment:combination | 2.21 | 2 | 0.33 |
| Infection treatment:test period | 0.23 | 1 | 0.63 |

**Supplementary Table S4: Post hoc tests results of the significant effect of the interaction between combination and test period on *Anopheles gambiae* activation rate.**

| **Pairwise** | **LRT** | **df** | ***P*** |
|---|---|---|---|
| Human-calf *vs* calf-control | 0.59 | 1 | 0.32 |
| Human-calf *vs* human-control | 7.79 | 1 | **0.02*** |
| Calf-control *vs* human-control | 3.71 | 1 | 0.11 |

**Supplementary Table S5: Effect of infection treatment, combination on *Anopheles arabiensis* mosquito activation rate.**

| Variable | LRT | df | p |
|---|---|---|---|
| **Test period 1 (oocyst stage)** | | | |
| Infection treatment | 0.58 | 1 | 0.45 |
| Combination | 10.34 | 2 | **0.005**** |
| Infection treatment: combination | 4.95 | 2 | 0.08 |
| **Test period 2 (sporozoite stage)** | | | |
| Infection treatment | 6.69 | 1 | **0.01**** |
| Combination | 6.61 | 2 | **0.04*** |
| Infection treatment:combination | 0.18 | 2 | 0.92 |

**Supplementary Table S6: Post hoc test result for the significant effect of combination on *Anopheles arabiensis* activation rate during test period 1**

| Pairwise | LRT | df | *P* |
|---|---|---|---|
| Human-calf vs calf-control | 1.79 | 1 | 0.18 |
| Human-calf vs human-control | 3.43 | 1 | 0.06 |
| Calf-control vs human-control | 9.96 | 1 | **0.002**** |

**Supplementary Table S7: Post hoc test results for the significant effect of combination on *Anopheles arabiensis* activation rate during test period 2.**

| Pairwise | LRT | df | *P* |
|---|---|---|---|
| Human_calf vs calf control | 0.01 | 1 | 0.90 |
| Human_calf vs human control | 4.97 | 1 | **0.03*** |
| Calf_control vs human control | 4.68 | 1 | **0.03*** |

**Supplementary Table S8: Effect of infection treatment and test period on *Anopheles coluzzii* host choice**

| Variable | LRT | df | *P* |
|---|---|---|---|
| Human_calf combination | | | |
| Infection treatment | 0.73 | 1 | 0.40 |
| Test period | 0.03 | 1 | 0.87 |
| Infection treatment:test period | 0.31 | 1 | 0.58 |
| Human_control combination | | | |
| Infection treatment | 1.24 | 1 | 0.27 |
| Test period | 0.02 | 1 | 0.89 |
| Infection treatment:test period | 0.62 | 1 | 0.43 |

**Supplementary Table S9: Effect of infection treatment, test period on *Anopheles gambiae* host choice**

| Variable | LRT | df | *P* |
|---|---|---|---|
| Human_calf combination | | | |
| Infection treatment | 0.12 | 1 | 0.73 |
| Test period | 1.96 | 1 | 0.16 |
| Infection treatment: test period | 0.47 | 1 | 0.49 |
| Human_control combination | | | |
| Infection treatment | 2.92 | 1 | 0.09 |
| Test period | 1.66 | 1 | 0.20 |
| Infection treatment: test period | 0.82 | 1 | 0.37 |
| Calf_control combination | | | |
| Infection treatment | 0.75 | 1 | 0.75 |
| Test period | 0.40 | 1 | 0.53 |
| Infection treatment:test period | 4.67 | 1 | **0.03*** |

**Supplementary Table S10: Effect of infection treatment on *Anopheles arabiensis* host choice during test period 1.**

| Variable | LRT | df | p |
|---|---|---|---|
| **Human-calf combination** | | | |
| Volunteer | 0.69 | 1 | 0.41 |
| Infection treatment | 0.87 | 1 | 0.35 |
| **Human-control combination** | | | |

| | | | |
|---|---|---|---|
| Infection treatment | 0.16 | 1 | 0.69 |
| **Calf-control combination** | | | |
| Infection treatment | 1.01 | 1 | 0.31 |

**Supplementary Table S11: Effect of infection treatment and volunteer on *Anopheles arabiensis* host choice during test period 2.**

| Variable | LRT | df | p |
|---|---|---|---|
| **Human-calf combination** | | | |
| Infection treatment | 0.36 | 1 | 0.55 |
| **Human-control combination** | | | |
| Infection treatment | 1.39 | 1 | 0.24 |
| **Calf-control combination** | | | |
| Infection treatment | 0.06 | 1 | 0.81 |

**GLMM models for all three groups of mosquitoes: exposed-infected, exposed-uninfected and unexposed.**

**Supplementary Table S12: Effect of infection status, combination and test period on *Anopheles coluzzii* mosquito activation rate.**

| Variable | LRT | df | *P* |
|---|---|---|---|
| Test period | 0.01 | 1 | 0.91 |
| Infection status | 1.12 | 2 | 0.57 |
| Combination | 3.09 | 1 | 0.08 |
| Infection status: combination | 0.27 | 2 | 0.88 |
| Infection status:test period | 2.17 | 2 | 0.34 |
| Test period:combination | 1.81 | 1 | 0.18 |

* $P < 0.05$, ** $P < 0.01$, *** $P < 0.001$

**Supplementary Table S13: Effect of infection status and test period on *Anopheles coluzzii* mosquito host choice for the two types of combination.**

| Variable | LRT | df | *P* |
|---|---|---|---|
| **Human-calf combination** | | | |
| Infection status | 1.56 | 2 | 0.46 |
| Test period | 0.54 | 1 | 0.46 |
| Infection status:test period | 0.35 | 2 | 0.84 |
| **Human-control combination** | | | |
| Infection status | 1.62 | 2 | 0.45 |
| Test period | 0.03 | 1 | 0.85 |
| Infection status:test period | 1.28 | 2 | 0.53 |

**Supplementary Table S14: Effect of infection status, volunteer, test period on *Anopheles gambiae* mosquito activation rate.**

| Variable | LRT | df | *P* |
|---|---|---|---|
| Infection status | 15.68 | 2 | **< 0.001***** |
| Combination | 15.66 | 2 | **<0.001***** |
| Test period | 3.96 | 1 | **0.046 *** |
| Infection status:combination | 10.87 | 4 | **0.03*** |
| Test period:combination | 6.78 | 2 | **0.03*** |
| Infection status:test period | 0.21 | 2 | 0.90 |

We did not run the model for the host choice due to insufficient data.

We found a significant effect of infection status effect on mosquito activation rate. The activation rate of exposed-uninfected females (12.06 ± 4.84 %, 21/174) was significantly lower than the activation rate of exposed-infected and uninfected control females (18.42 ± 3.18%, 105/570 and 19.25 ± 2.99%, 129/670, respectively; table S14a). This may

suggest an energy cost of resisting infection whereby females that invested in immunity and resisted the infection had less energy to invest in host-seeking. In addition, there was a significant infection status by combination interaction although post-hoc test for pairwise interactions showed non-significant differences (see table S14b & c).

**Supplementary Table S14a: Post hoc tests results of the significant effect of the infection status on *Anopheles gambiae* activation rate.**

| **Pairwise** | **LRT** | **df** | ***P*** |
|---|---|---|---|
| Control – exposed infected | 0.26 | 1 | 0.55 |
| Control – exposed uninfected | 9.59 | 1 | **0.006**** |
| Exposed infected – Exposed uninfected | 8.11 | 1 | **0.008**** |

**Supplementary Table S14b: Post hoc tests results of the significant effect of the interaction between combination and infection status on *Anopheles gambiae* activation rate.**

| **Pairwise interaction** | **LRT** | **df** | ***P*** |
|---|---|---|---|
| Ctrl-Inf * CO-HC | 0.96 | 1 | 1 |
| Ctrl-Uninf * CO-HC | 2.89 | 1 | 0.62 |
| Inf-Uninf * CO-HC | 1.21 | 1 | 1 |
| Ctrl-Inf * CO-HO | 0.26 | 1 | 1 |
| Ctrl-Uninf * CO-HO | 4.24 | 1 | 0.32 |
| Inf-Uninf * CO-HO | 4.93 | 1 | 0.24 |
| Ctrl-Inf * HC-HO | 2.12 | 1 | 0.87 |
| Ctrl-Uninf * HC-HO | 0.90 | 1 | 1 |
| Inf-Uninf * HC-HO | 2.01 | 1 | 0.87 |

Ctrl: Control; Inf: Exposed infected; Uninf: Exposed uninfected; HO: Human odour – Outdoor air, CO: Calf odour– Outdoor air; HC : Human odour-Calf odour

**Supplementary Table S14c: Post hoc tests results of the significant effect of the interaction between combination and infection status on *Anopheles gambiae* activation rate.**

| **Pairwise** | **LRT** | **df** | ***P*** |
|---|---|---|---|
| CO-HC * T1-T2 | 0.20 | 1 | 0.66 |
| CO-HO * T1-T2 | 4.38 | 1 | 0.07 |
| HC-HO * T1-T2 | 5.57 | 1 | 0.054 |

CO: Calf – Outdoor air; HC: Human odour – Calf odour; HO: Human odour – Outdoor air; T1: Testing period 1; T2: Testing period 2.

**Supplementary Table S15: Effect of infection status, volunteer and combination on *Anopheles arabiensis* mosquito activation rate.**

| Variable | LRT | df | *P* |
|---|---|---|---|
| **Test period 1 (oocyst stage)** | | | |
| Infection status | 0.55 | 2 | 0.76 |
| Combination | 14.92 | 2 | **<0.001*** ** |
| Infection status:combination | 5.46 | 4 | 0.24 |
| **Test period 2 (sporozoite stage)** | | | |
| Infection status | 9.45 | 2 | **0.009** ** |
| Combination | 11.38 | 2 | **0.003** ** |
| Infection status:combination | 1.59 | 4 | 0.81 |

We did not run the model for the host choice due to insufficient data.
